# Conditional Knockout of Telomerase Reverse Transcriptase in Mesenchymal Cells Impairs Mouse Pulmonary Fibrosis

**DOI:** 10.1371/journal.pone.0142547

**Published:** 2015-11-10

**Authors:** Tianju Liu, Hongfeng Yu, Lin Ding, Zhe Wu, Francina Gonzalez De Los Santos, Jianhua Liu, Matthew Ullenbruch, Biao Hu, Vanessa Martins, Sem H. Phan

**Affiliations:** 1 Department of Pathology, University of Michigan School of Medicine, Ann Arbor, Michigan, United States of America; 2 Faculty of Medicine, University of São Paulo, São Paulo, Brazil; University of Allabama at Birmingham, UNITED STATES

## Abstract

Telomerase is typically expressed in cellular populations capable of extended replication, such as germ cells, tumor cells, and stem cells, but is also induced in tissue injury, repair and fibrosis. Its catalytic component, telomerase reverse transcriptase (TERT) is induced in lung fibroblasts from patients with fibrotic interstitial lung disease and in rodents with bleomycin-induced pulmonary fibrosis. To evaluate the fibroblast specific role of TERT in pulmonary fibrosis, transgenic mice bearing a floxed TERT allele were generated, and then crossed with an inducible collagen α2(I)-Cre mouse line to generate fibroblast specific TERT conditional knockout mice. TERT-specific deficiency in mesenchymal cells caused attenuation of pulmonary fibrosis as manifested by reduced lung hydroxyproline content, type I collagen and α-smooth muscle actin mRNA levels. The TERT-deficient mouse lung fibroblasts displayed decreased cell proliferative capacity and higher susceptibility to induced apoptosis compared with control cells. Additionally TERT deficiency was associated with heightened α-smooth muscle actin expression indicative of myofibroblast differentiation. However the impairment of cell proliferation and increased susceptibility to apoptosis would cause a reduction in the myofibroblast progenitor population necessary to mount a successful myofibroblast-dependent fibrotic response. These findings identified a key role for TERT in fibroblast proliferation and survival essential for pulmonary fibrosis.

## Introduction

Telomerase is a ribonucleoprotein complex comprised of a catalytic component, telomerase reverse transcriptase (TERT) and an RNA template (TR). It has a well-established function of adding telomeric DNA forming telomeres to the ends of linear chromosomes [1]. TERT, the protein component of telomerase, is expressed in stem cells and progenitor cells in normal tissues but is undetectable in normal adult human somatic cells [2, 3] although it can be induced in certain cells upon appropriate stimulation [4, 5]. Telomerase activity is widely increased in many germ cells and cancerous cells [6–8]. The role of telomerase in telomere maintenance and the importance of telomeres in DNA replication implicate its intimate involvement in cell proliferation, aging and senescence [9–11]. Telomere shortening due to telomerase deficiency and/or other causes is associated with multiple conditions and diseases, including pulmonary fibrosis [12, 13]. However the mechanism is not always clear as to how telomere shortening dependent or independent of telomerase deficiency could result in chronic fibrotic disease for instance. Moreover, in addition to this canonical telomere maintenance function, there is mounting evidence that TERT has non-canonical functions of potential import in development, cell differentiation and certain disease processes [14, 15]. For example TERT in mice (mTERT) promotes hair follicle stem cell proliferation in a mechanism independent of the TR [14]. It enhances keratinocyte proliferation and activates resting hair follicle stem cell through transcriptional regulation of a developmental program associated with the Myc and Wnt pathways [15, 16]. There is also evidence that mammalian TERT has extranuclear function in mitochondria [17, 18]. Mammalian TERT contains a nuclear export signal[19] as well as a putative mitochondrial targeting sequence [20] to guide its oxidative stress induced relocation from the nucleus to mitochondria where its function is not related to maintenance of telomeres [21]. A further complication is that selective telomerase expression and/or activity in different cell types can have different impact on disease processes, such as chronic fibrosis.

A growing body of evidence reveals that TERT expression is transiently induced in tissue injury, repair and fibrosis [10, 22, 23]. Selective over-expression of TERT in dermal basal keratinocytes results in increased skin wound healing rate, in addition to increasing susceptibility to tumor formation[10]. This may reflect a proliferative advantage of high TERT/telomerase expressing tissues in response to proliferative signals associated with wound healing. In a humanized mouse model, human TERT (hTERT) promoter activity is not active in resting liver, but in response to liver injury it is markedly activated in proliferating hepatocytes during liver regeneration with potential involvement of E2F2 and E2F7 transcription factors, thus implicating hTERT as a potential factor underlying the regenerative capacity of human liver [23]. Telomerase is transiently increased in lung injury, induced by bleomycin (BLM), hypoxia or silica [4, 5, 24]. Telomerase induction in lung fibroblasts from BLM-treated mice is accompanied by increased TERT expression but without significant effect on telomere length. In contrast, TERT deficiency reduces myofibroblast differentiation and impairs lung fibrosis, which is partially reversed by transplantation with wild type (WT) bone marrow (BM) resulting in restoration of telomerase induction in BLM-injured lung. These findings implicate the importance of TERT during lung fibrosis [25]. Similarly, marked induction of telomerase activity and TERT expression are found in a murine pulmonary hypertension (PH) model as well as in lungs from patients with idiopathic PH [26]. Moreover TERT deficient mice develop less severe PH with diminished proliferation of vascular smooth muscle cells without affecting telomere length. In contrast, mutant TERT, TR and/or shortened telomeres are suggested as risk factors for IPF [12, 13, 27, 28]. In a small subset of cases with familial pulmonary fibrosis, other rare mutations in telomere associated genes have been subsequently reported, including TINF1, RTEl1, PARN and DKC1 [29–32]. However another study with a different patient population reveals <6% of IPF or hypersensitivity pneumonitis patients exhibited shortened telomeres in peripheral blood leukocytes and <4% in lung fibroblasts [33]. In contrast, about 66% of lung fibroblast samples from IPF patients express telomerase with a much lower percentage in those from hypersensitivity pneumonitis patients, thus suggesting association of fibrotic interstitial lung disease with induction of telomerase in lung fibroblasts without impact on telomere length. These different studies would seem to suggest the potential importance of telomere shortening on the one hand, and the induction of telomerase on the other. This apparent conflict could be due to induction of telomerase in different cell types, which might have diverse effects on the pathogenesis of chronic fibrotic lung diseases. For instance, regeneration and re-epithelialization of the alveolar epithelium in lung repair may benefit from induction of telomerase but may be impaired by telomere shortening, while having similar effects on fibroblasts. However while epithelial regeneration or proliferation is beneficial for repair, fibroblast proliferation is detrimental by promotion of fibrosis instead of proper healing. It is unclear if the induction of telomerase in fibrosis is restricted to lung fibroblasts and/or the role of this induction in fibrosis is restricted to fibroblasts only. While TERT knockout mice are available to study its overall role(s) globally in vivo, the divergent roles of TERT in different cell types vis-à-vis disease processes in different organ systems and tissues necessitate a conditional cell specific approach to study effects of its deficiency.

## Materials and Methods

### Generation of TERT conditional knockout mice

**Fig 1:**
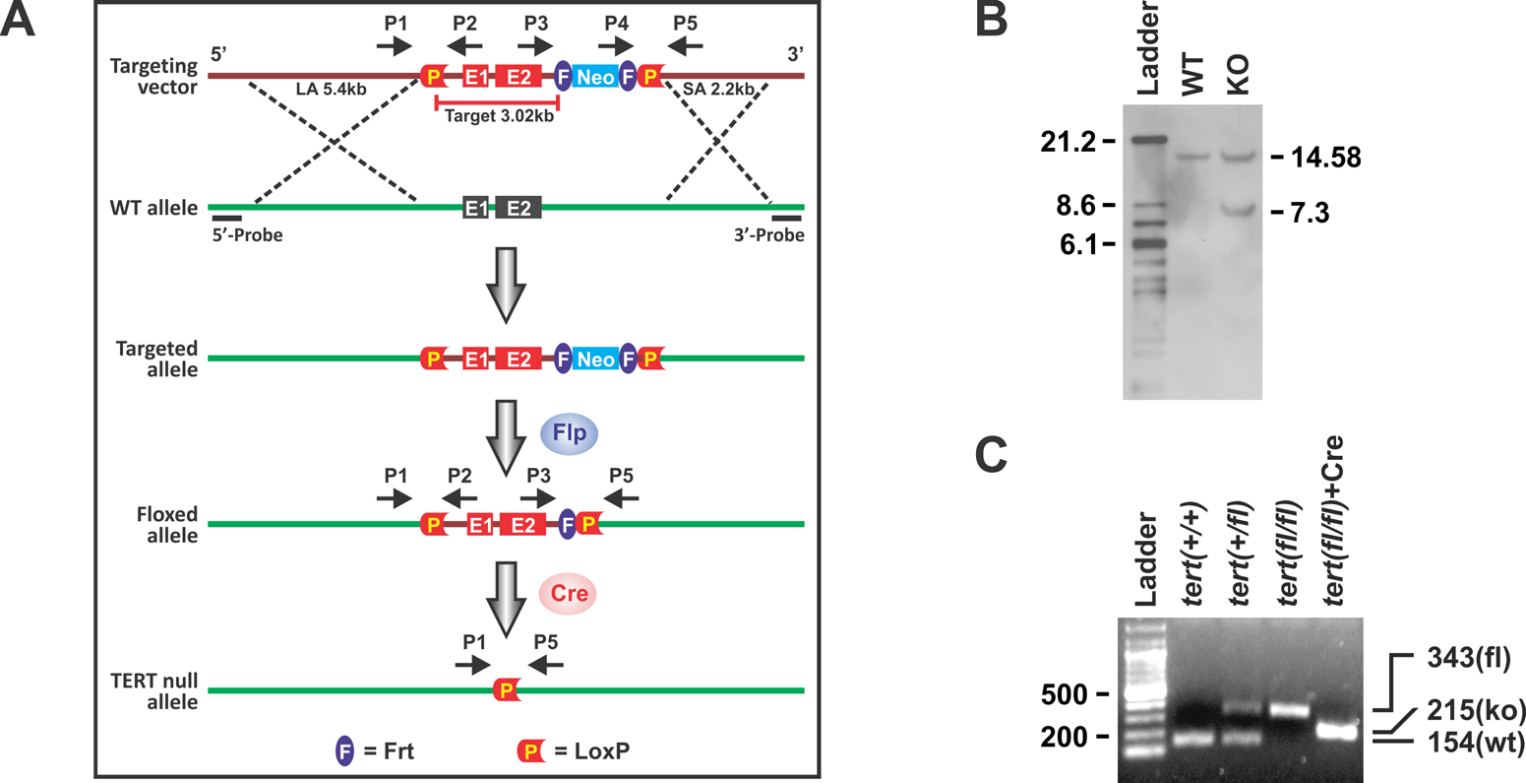
Generation and verification of the TERT CKO mice. (A) Schematic gene-targeting map of TERT gene. The construction of TERT floxed and TERT CKO alleles are shown before and after tamoxifen treatment. TERT gene exon 1–2 was floxed after recombination between WT and targeted alleles. Primers pairs P1/P2 and P3/P5 were used to detect floxed TERT alleles. Primer pair P1/P5 was used to detected the TERT gene excision by Cre. (B) Representative Southern blot analysis. The ES clone with homologous recombination was digested with the restriction enzyme Bsr GI followed by Southern blotting with Dig-labeled 5’ probe as shown in (A). The detected WT and targeted alleles are 14.58 and 7.3 kb, respectively. (C) PCR genotyping using genomic DNA from mouse tails. The PCR fragments for WT was 154bp, for TERT fl/+ they were 154 and 343 bp, for TERT *fl/fl* it was 343 bp, and for TERT CKO it was 215bp after tamoxifen-induced Cre excision. “Ladder” referred to 100 bp DNA ladder.

A 10.65 kb region from C57BL/6 BAC clone (pSP72 as backbone vector, Promega, Fitchburg, WI) was used to construct the targeting vector. A loxP/FRT flanked *neomycin* cassette was inserted on the 3’ side of exon 2 and a single loxP site was inserted approximately 1 kb 5’ of exon 1. The total size of the targeting construct (including vector backbone and neomycin cassette) was 14.75 kb with 3.02 kb comprising the target region (Fig 1A). The TERT targeting vector was electroporated into C57BL/6 ES cell Bruce 4. Southern blotting with the digoxin (Dig)-labeled probe was conducted to screen ES cell clones with successful homologous recombination. WT and floxed alleles generated 14.58 kb and 7.3 kb fragments, respectively (Fig 1B).Three of the ES cell clones were used for blastocyst microinjections. After confirming germline transmission, the chimeras were crossed with FLP mice (The Jackson Laboratory) to remove the *neomycin* cassette. The resulting homozygous mice with LoxP sites as indicated in the TERT gene are hereafter referred to as floxed TERT (or TERT*fl/fl*) mice. Floxed TERT mice were then crossed with C57BL/6J-Tg[*Col1α2-Cre-ER(T)*] (Cre+/-) mice bearing a tamoxifen-inducible Cre-recombinase under the control of a regulatory sequence from the α2(I) collagen gene (gift of Dr. Benoit deCrombrugghe, University of Texas MD Anderson Cancer Center) to generate TERT *fl/fl*,Cre+/- mice. Tamoxifen-induced Cre excision resulted in the conditional type I collagen expressing mesenchymal (‘fibroblast’) specific TERT CKO. A new 215 bp PCR fragment was generated after Cre excision with primers 1 and 5 (P1 and P5 in Fig 1A).Genotyping was done by PCR and a representative result is shown (Fig 1C). All primers are available upon request.

### Induction of mouse pulmonary fibrosis model

Pulmonary fibrosis was induced in TERT CKO or relevant control Cre+/- (for simplicity referred to as WT) mice (n = 3~5) by endotracheal BLM (Blenoxane, Mead Johnson, NJ) injection at a dose of 2 U/kg bodyweight as before [34]. To induce TERT deficiency in fibroblasts, 1 mg/mouse of 4-OHT (Sigma, St. Louis, MO) was given by i.p. injection daily for 8 consecutive days prior to BLM administration on day 0. Daily tamoxifen injection (1 mg/mouse) was continued after BLM injection until the day of sacrifice as before [35]. Animals were randomly assigned to the indicated treatment groups. Twenty-one days after BLM treatment, the mice were sacrificed and the lungs were harvested rapidly for tissue RNA/protein preparation, fibroblast isolation, hydroxyproline (HYP) assay and histopathological examination. All animal studies were reviewed and approved by the Committee on Use and Care of Animals at the University of Michigan.

### Cell isolation and treatments

MLF were isolated using a digestion cocktail containing collagenase III and DNase I (Worthington Biochemical Crop., Lakewood, NJ), and maintained in DMEM supplemented with 10% plasma-derived fetal bovine serum (PDS; Animal Technologies, Tyler, TX), 10 ng/ml of EGF and 5 ng/ml of PDGF (R&D systems, Inc. Minneapolis, MN) as before [36]. MLF at passages 1–5 were used in the indicated experiments. Where indicated CD11b+ cells were depleted from the MLF cultures using MACS system with CD11b microbeads (Miltenyi Biotec., Auburn, CA). To induce Cre expression MLF were transduced with 100 MOI of Ad5CMVCre-eGFP (AdCre; Viral Vector Core Facility, University of Iowa, IA) or by treatment with 5 μM of 4-OHT in the case of cells from TERT CKO mice. Primary AEC II were isolated as previously described [37]. Briefly, lungs were instilled with dispase II (Roche Diagnostics, Indianapolis, IN) followed by low-melt agarose (Sigma), and digested for 45 minutes. Lungs were then dissected and treated with DNase. The cell suspensions were negatively selected for CD16/32 and CD45 expressing cells by MACS separation system followed by further negative selection for non-adherent cells by incubation on the petri dishes. The AEC II were then plated and cultured on fibronectin-coated plates (BD Biosciences, San Jose, CA) before use. T and B lymphocytes were isolated and purified from thymus and spleen, respectively using CD90.2 (for T-cells) and CD220 (for B-cells) microbeads (Miltenyi Biotec.). Human normal foreskin fibroblast BJ and its hTERT-immortalized counterpart BJ 5ta were purchased from ATCC (Manassas, VA), and maintained in DMEM supplemented with 10% of fetal bovine serum (Sigma).

### qRT-PCR and telomerase activity assay

Total RNA was isolated using Trizol (Invitrogen). The Taqman primers for mouse procollagen I, α-SMA, TERT and 18s RNA were purchased from Life Technologies. 18s RNA was used as reference to normalize the input RNA. One-step real-time RT-PCR was performed on a GeneAmp 7500 Sequence Detection System (Applied Biosystems). Results were expressed as 2^-ΔΔCT^ using the indicated control group as calibrator.

Telomerase activity in the lung tissue or isolated MLF was assayed using a telomerase PCR ELISA kit (Roche) in accordance with the manufacturer’s protocol. The heat-inactivated tissue or cell lysates were used for the negative controls.

### Cell proliferation and apoptosis assay

Fibroblasts isolated from 4-OHT-treated WT or TERT CKO lungs at passage 1 were seeded into 96-well plates (5×10^3^ cells/well). After 24 hours, 10 ng/ml of PDGF (R & D systems) was added and cultured for the indicated times up to 96 hours. The cell number was counted using a Z2 Particle Count and Size Analyzer (Beckman Coulter, Inc., Indianapolis, IN). Where indicated, 10 μl of WST-1 reagent (Roche) was added 48 hours after PDGF treatment for measuring cell proliferation by WST-1 assay as described before [25]. Apoptosis assay was performed in WT or TERT KO MLF treated with 5 ng/ml TNFα and 500 ng/ml CHX using TACS AnnexinV-FITC kit (R & D systems) and propidium iodide (PI) as described before [25]. Analysis was undertaken using a FACS Caliber flow cytometer (BD Biosciences). Apoptotic cells were identified as an annexin V+/PI- population.

### Hydroxyproline assay

Lung collagen deposition was estimated by measuring the hydroxyproline (HYP) content of whole lung homogenates using Hydroxyproline assay kit (Sigma) in accordance with the manufacturer’s protocol. The results were expressed as μg HYP per lung.

### Histopathological analysis

The lungs were inflated by intratracheal perfusion and fixed for 24 h with 10% buffered formaldehyde. Lung tissue was then paraffin embedded, sectioned, and stained with hematoxylin and eosin (H&E).

### Statistics

All data were expressed as mean ± SD unless otherwise indicated. Differences between means of various treatment and control groups were assessed for statistical significance by ANOVA followed by *post hoc* analysis using Scheffé's test. A P value < 0.05 was considered to indicate statistical significance.

## Results

### Characterization of floxed TERT mice and Cre-induced TERT CKO mice

**Fig 2:**
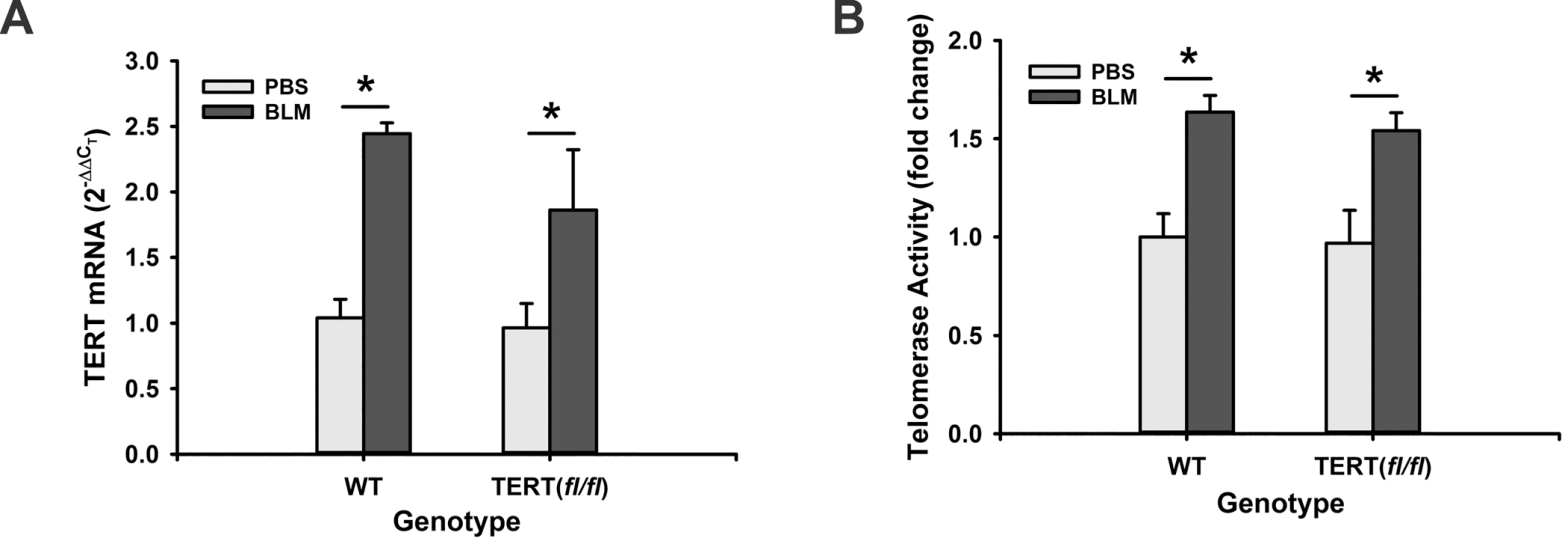
TERT and telomerase in floxed TERT vs WT mice. Total RNA or protein lysates were prepared from the lung tissues and MLF from BLM or PBS-injected TERT *fl/fl* or WT mice. TERT gene expression was analyzed by qRT-PCR and expressed as 2^-ΔΔCT^ (n = 3) in (A), and the telomerase activities were detected by TRAP-ELISA kit and expressed as fold change over their PBS control, respectively (B). n = 3. *, P < 0.05.

**Fig 3:**
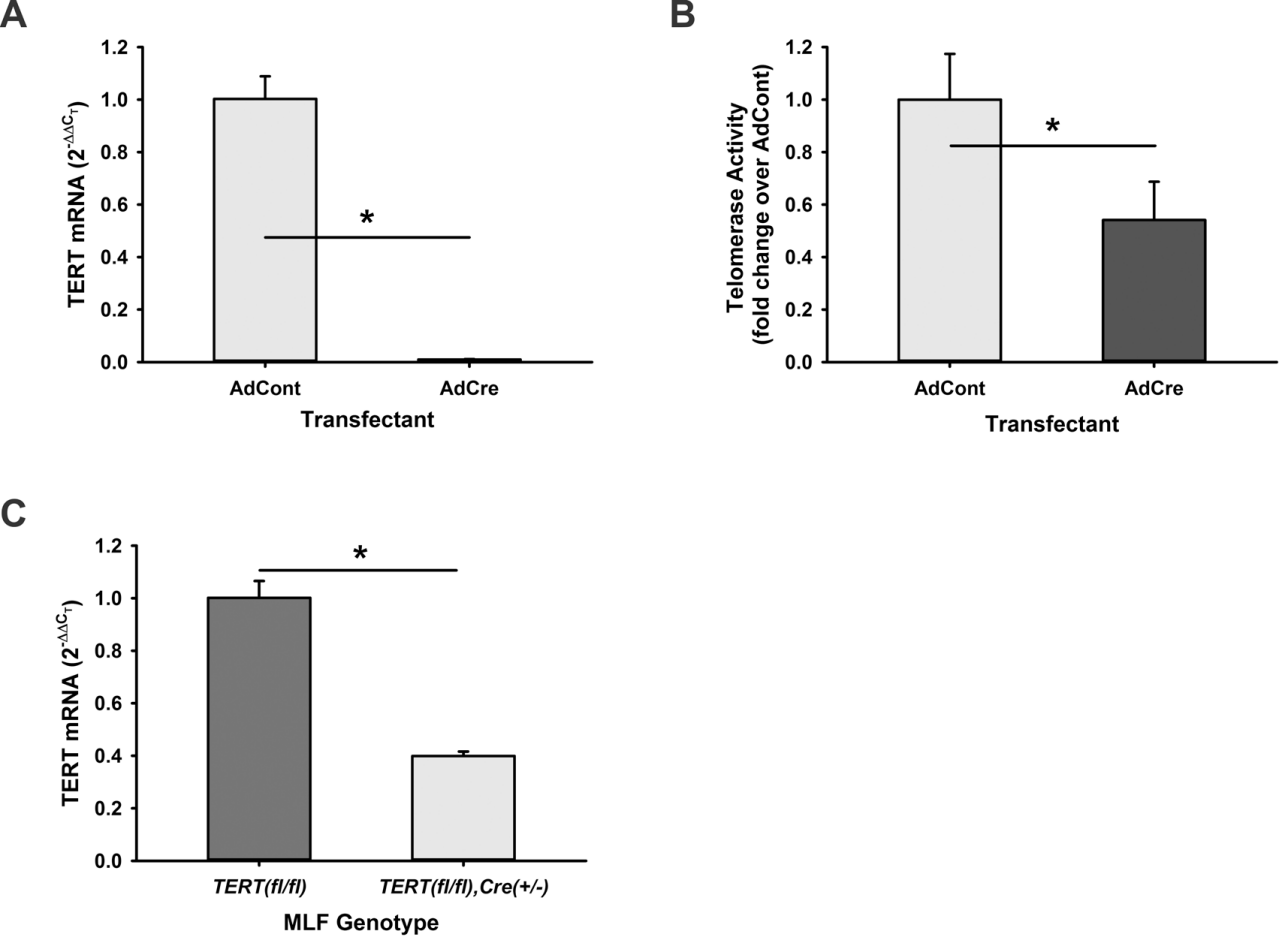
The in vitro excision of MLF TERT by Cre activation. The MLF were isolated from TERT *fl/fl* mice, and then transduced with 100 MOI of AdCre vector or AdGFP control vector. Six days after transduction, TERT mRNA (A) and telomerase (B) were analyzed, respectively, as described in the legend of Fig 2. n = 3. (C) The MLF from TERT *fl/fl* or TERT *fl/fl*/,Cre+/- mice were treated with 5 μM of 4-OHT in vitro at the same time for 6 days, and TERT mRNA was analyzed. n = 3. *, P < 0.05.

**Fig 4:**
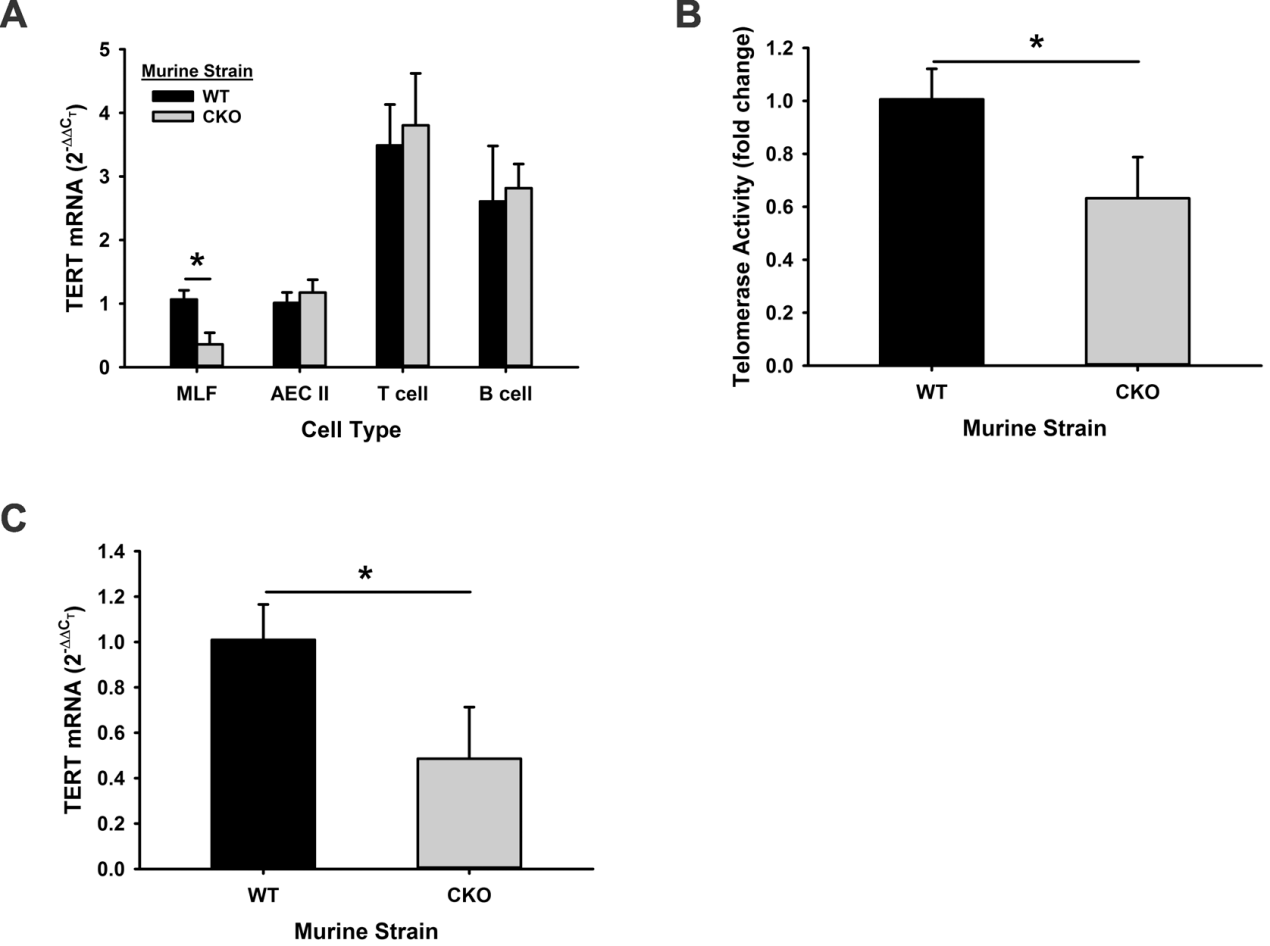
Selective knockout of TERT gene in TERT CKO mice. (A) MLF, AEC II, thymic T, and splenic B cells were isolated from TERT CKO and WT mice. TERT mRNA expression was examined in these cells. n = 3 for each. (B) Telomerase activity was measured in the MLF from TERT CKO and control mice. n = 3. (C) The TERT mRNA expressions in TERT CKO and control lung tissues are shown. n = 3. *, P < 0.05.

Floxed TERT (TERT *fl/fl*) mice were generated on C57Bl/6 background as described in the Methods. There was no physical difference between the wild type (WT) and floxed mice with respect to general appearance and body weight gain or growth rate. To examine whether or not the insertion of LoxP sites influenced the TERT gene integrity, TERT gene expression and telomerase activity were first examined in mouse lung fibroblasts (MLF). The results showed that TERT gene expression in TERT *fl/fl* MLF was not altered compared with MLF from WT animals. Since the TERT mRNA level is elevated in BLM-induced pulmonary fibrosis, lung TERT expression was also evaluated in BLM-treated TERT *fl/*fl mice. Comparable induction by BLM was observed between TERT *fl/fl* and WT mice in both isolated MLF and lung tissue (Fig 2A). There was an approximate 2-fold induction for TERT mRNA, which was accompanied with a >60% increase for telomerase activity in MLF and lung tissue (Fig 2B). These findings indicated that TERT gene expression and function were intact in the TERT *fl/fl* mice and remained capable of responding to regulatory signals in response to lung injury and fibrosis. Next, the effect of Cre recombinase expression on TERT gene expression in MLF from TERT*fl/fl* mice was investigated in vitro. The results showed that Cre adenovirus transfection dramatically inhibited TERT mRNA expression to almost undetectable levels, while the control adenovirus did not affect TERT gene expression compared with untransduced TERT *fl/fl* fibroblast (Fig 3A). This ablation of TERT expression resulted in a significant reduction in telomerase activity (Fig 3B). The reduction of TERT expression was also noted in MLF isolated from the TERT *fl/fl*,Cre+/- mice upon in vitro treatment with 5μM 4-hydroxy-tamoxifen (4-OHT) (Fig 3C), although the 4-OHT effect was not as complete as that attained by induced Cre expression using Cre adenovirus. To confirm that TERT gene is selectively knockout in collagen I-expressing mesenchymal cells, TERT gene expression was evaluated in mouse AEC II, T cells and B cells isolated from thymus and spleen, respectively, of TERT CKO mice. While the >70% inhibition of TERT gene expression was induced in MLF as expected, no alteration was observed in AEC II, T and B cells from TERT CKO mice after 7 days of tamoxifen treatment (Fig 4A). These findings confirmed the cell specificity of induced TERT gene deficiency in mesenchymal cells/MLF. Consistent with the TERT mRNA reduction in MLF, the telomerase activity in CD11b depleted MLF was also significantly inhibited (Fig 4B). This significant reduction in MLF TERT gene expression was also noted in lung tissue from TERT CKO mice compared with that in the control lungs (Fig 4C). The reduction in TERT gene and telomerase activity in MLF from TERT CKO mice was unchanged with a further 3 days (to a total of 10 days) of 4-OHT treatment (data not shown). These results confirmed that the TERT gene could be successfully excised by tamoxifen-induced Cre recombinase specifically in collagen I-expressing mesenchymal cells, although the extent was incomplete (<30% residual for mRNA).

#### The effect of mesenchymal specific TERT deficiency on BLM-induced pulmonary fibrosis

**Fig 5:**
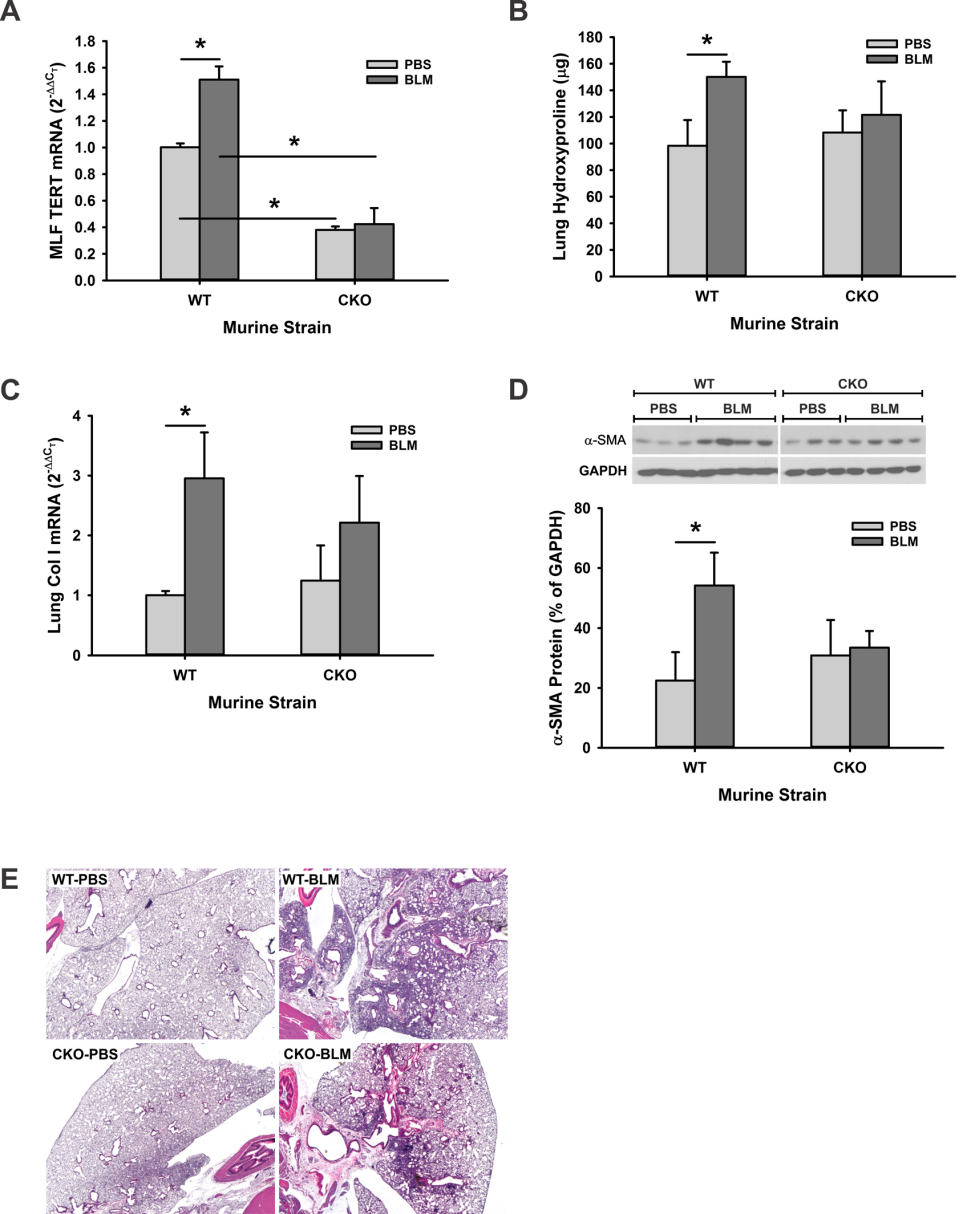
The impairment of pulmonary fibrosis in TERT CKO mice. (A) The MLF were isolated from TERT CKO or WT mice at 21 days after BLM injection, and analyzed for TERT mRNA by qRT-PCR. The expression was expressed as the fold change of the level in PBS-treated WT MLF. n = 3–5 mice per group. (B) The lungs from TERT CKO and control mice were homogenized at day 21 after BLM treatment, and measured for whole lung collagen content by HYP assay. n = 3–5 mice per group. (C) Lung tissue RNA extracted from the indicated murine strains was also analyzed for type I collagen mRNA by qRT-PCR. n = 3–5 mice per group. (D) Lung tissue lysates were prepared by RIPA buffer, and analyzed for α-SMA protein in the indicated murine strain by Western blotting (top panel). Quantitative data was normalized by the internal control GAPDH, and shown as the percentage of the GAPDH signals (bottom panel). n = 3–4 mice per group. (E) Representative H & E stained lung tissue sections at day 21after BLM treatment are shown. Original magnification × 20. *, P < 0.05.

We have previously shown that TERT is induced MLF and required for BLM-induced pulmonary fibrosis using TERT traditional KO mice [25]. However it is not clear whether TERT/telomerase in the different cell types play similar or diverse roles in fibrosis. To specifically determine the importance of TERT in the mesenchymal compartment vs that in epithelial and immune/inflammatory cell compartments, pulmonary fibrosis was induced in TERT CKO and control mice by endotracheal injection of BLM. TERT gene expression was first examined in both isolated MLF and lung tissues to confirm the TERT ablation after tamoxifen treatment. The level of TERT mRNA expression in MLF isolated from control (PBS-treated) TERT CKO mice was ~38% of that in MLF from control WT mice (Fig 5A). While MLF TERT mRNA was significantly increased upon BLM treatment of WT mice, it was not altered in TERT CKO mice, resulting in a >70% inhibition of TERT mRNA in the cells from BLM treated TERT CKO mice. Analysis of total lung collagen content by hydroxyproline (HYP) assay at 21 days post BLM injection revealed similar levels of HYP in lungs of control WT and TERT CKO mice (Fig 5B). However while BLM treatment caused the expected significant increase of HYP in lungs of WT mice, this effect essentially vanished in the TERT CKO mice, although the absolute value of the BLM-induced HYP in TERT CKO was not statistically different from that in BLM-treated WT lungs (P = 0.05). Consistent with this reduction in BLM-induced increase in TERT CKO lung HYP content lung the increase in WT lung type I collagen gene expression was similarly suppressed in the TERT CKO lungs (Fig 5C). Moreover the > BLM-induced 2-fold stimulation of α-smooth muscle actin (α-SMA) protein expression in WT lungs was essentially abolished in TERT CKO lungs (Fig 5D). Finally, histopathological assessment revealed that TERT CKO mice displayed less extensive fibrosis compared with the more diffuse fibrotic lesions affecting larger areas in lungs of WT mice (Fig 5E). Taken together, the fibroblast/mesenchymal-specific TERT deficiency resulted in impaired pulmonary fibrosis.

### The role of TERT in the cellular process of proliferation, survival and differentiation

**Fig 6:**
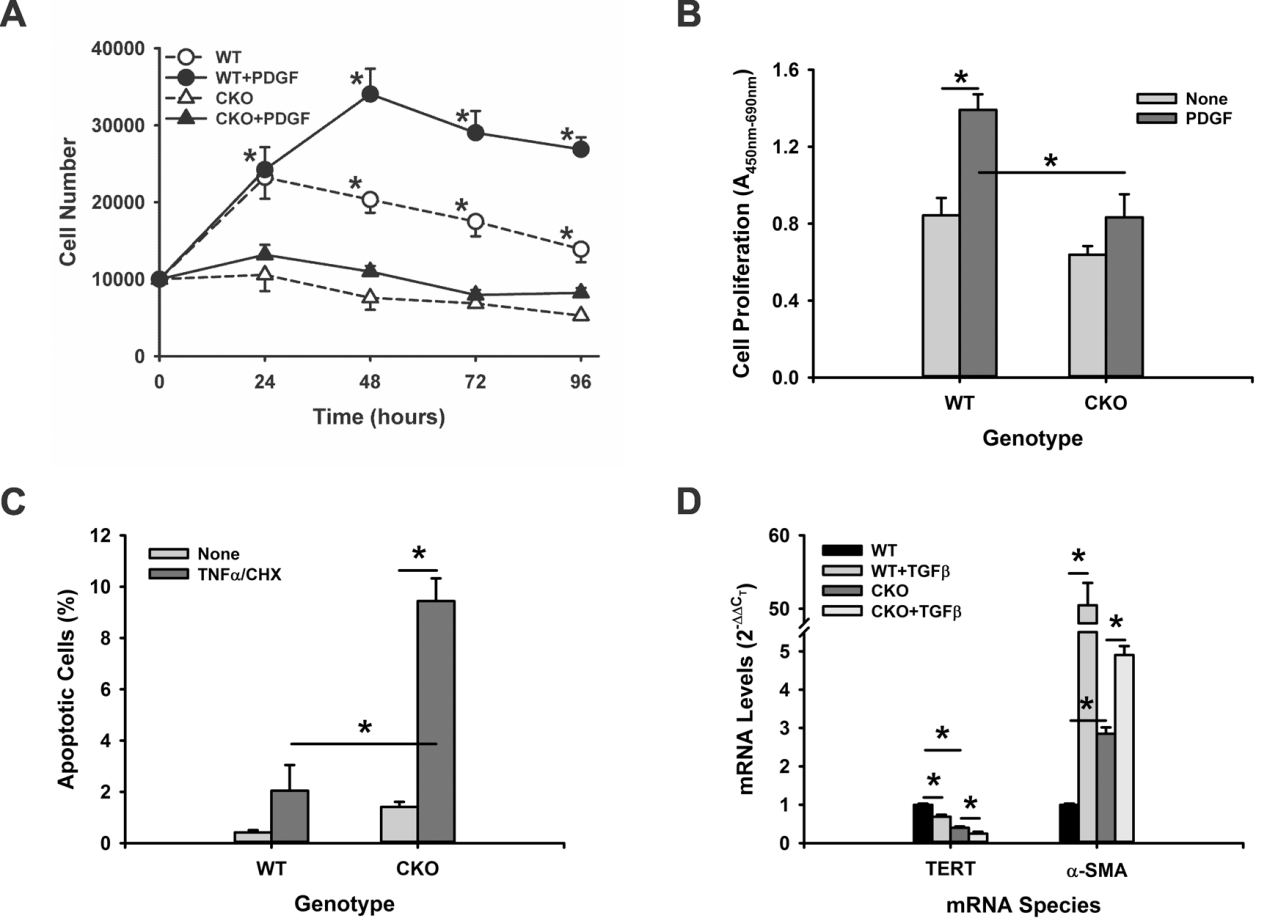
Effects of TERT CKO on MLF proliferation, apoptosis and differentiation. (A) The MLF were isolated from TERT CKO and control mice, and analyzed for proliferation by counting the cell numbers at the indicated time points. n = 5. (B) MLF proliferation by WST-1 assay at 48 hours after PDGF treatment. n = 6. (C) Apoptosis was also detected in MLF by annexin V-FITC/PI staining and flow cytometry without and with TNF-α/CHX treatment. The FITC+/PI- cells were referred to as the apoptotic cells. n = 3. (D) MLF isolated from TERT CKO or control mice were cultured and analyzed for TERT and a-SMA mRNA expression after treatment with buffer only or with TGF-β1 for 48 hours. The mRNA expression levels were expressed as the fold changes over the WT MLF for each gene. n = 3. *, P < 0.05.

Given the multiple roles of TERT, including non-canonical effects unrelated to telomere maintenance [16, 38, 39] and in myofibroblast differentiation [25], its role in mesenchymal cells in fibrosis require further exploration. Specifically, to seek out a potential mechanism for the impaired fibrosis in TERT CKO mice, the direct effects of TERT on cell proliferation, apoptosis, and differentiation were investigated. MLF from TERT CKO or WT mice were treated with PDGF and then analyzed for cell proliferation. WT cells doubled in 24 hours of culture, which were further significantly increased at 48 hours or later (up to 96 hours) upon treatment with PDGF (Fig 6A). In contrast, TERT CKO cells failed to proliferate in the absence or presence of PDGF. The TERT effect on cell proliferation was confirmed by WST-1 assay at 48 hours after PDGF stimulation (Fig 6B).

The effect on MLF susceptibility to apoptosis was then determined in TERT deficient MLF by evaluating the response to a known apoptotic stimulus, TNF-α combined with cycloheximide (CHX). Analysis of the treated cells by flow cytometry showed that in the absence of stimuli, WT MLF displayed a low apoptotic rate of 0.42%, which was >3-fold elevated but not statistically significant in TERT deficient MLF. However TNF-α/CHX treatment caused a significantly higher apoptosis rate (>4-fold) in TERT deficient MLF than in WT MLF (not statistically significant) when compared to their respective untreated controls (Fig 6C). Thus in addition to impaired proliferation TERT deficient MLF were more susceptible to apoptosis.

**Fig 7:**
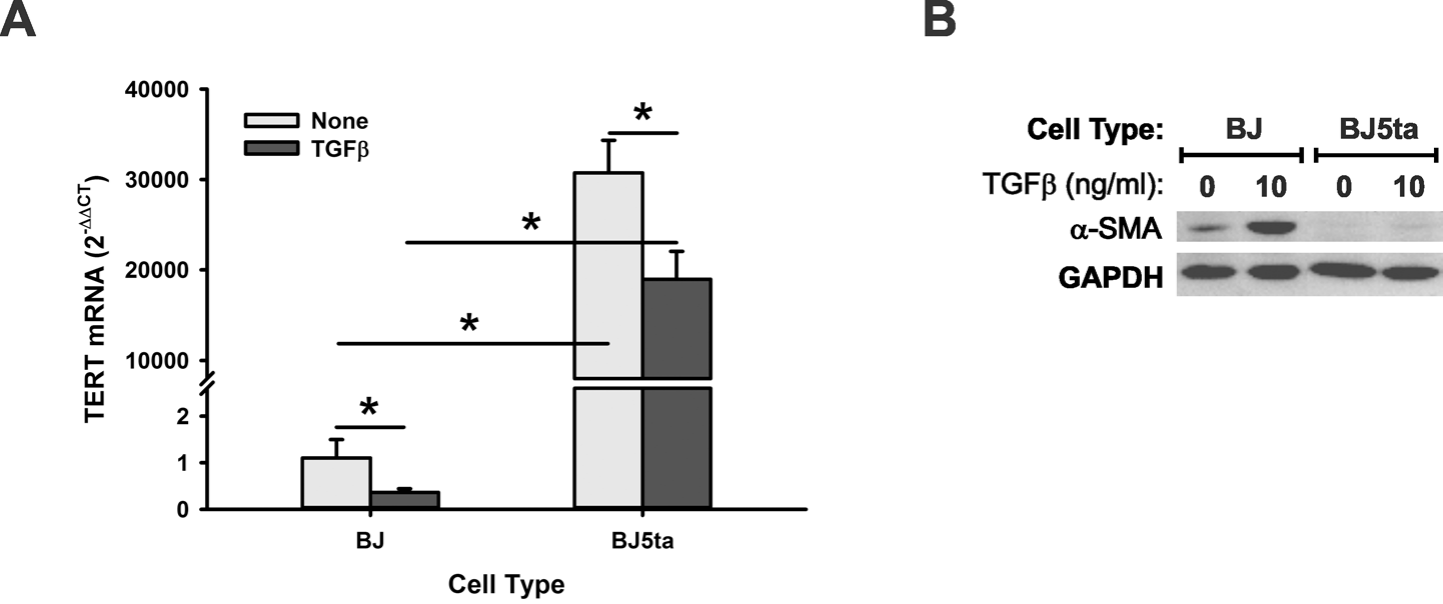
Effect of TERT overexpression on α-SMA expression. BJ and BJ 5ta fibroblasts were plated in 6-well plates. The cells were starved with DMEM supplemented with 0.5% FBS for 4 hours before TGF-β1 treatment for an additional 24 for mRNA or 48 hours for protein analysis. (A) BJ and BJ5ta cells were analyzed for TERT mRNA by qRT-PCR. n = 3. *, P < 0.05. (B) The cell lysates were harvested in RIPA buffer, and analyzed for α-SMA protein expression by Western blotting. A representative blot was shown. The GAPDH was used as internal control.

Finally, the effect of TERT deficiency on myofibroblast differentiation was analyzed. The expression level of α-SMA, a key marker of myofibroblast differentiation, was assessed in MLF from WT or TERT CKO mice. While the TERT mRNA was significantly decreased by >2-fold in MLF isolated from TERT CKO mice vs. WT mice, the α-SMA mRNA was significantly higher (2.8-fold increase) in TERT CKO MLF compared to that in WT MLF (Fig 6D). TGF-β1 treatment caused a further reduction in TERT mRNA but caused a further significant increase in α-SMA mRNA in TERT CKO cells. This inverse relationship between TERT and α-SMA expression was similarly observed in comparing BJ human foreskin fibroblasts to the BJ5ta fibroblasts with > 30,000-fold higher level of TERT mRNA due to stable transfection with hTERT (Fig 7A). While the expression of α-SMA could be easily detected by western blotting in BJ fibroblasts, it was barely detectable in BJ5ta fibroblasts with overexpressed TERT. Although TGF-β1 increased α-SMA levels in both BJ and BJ5ta cells, the increase in telomerase deficient BJ cells was more substantial (Fig 7B). Taken together TERT had the potential property of controlling cell proliferation, fate/differentiation, and susceptibility to apoptosis, which might mediate its mesenchymal cell specific role in pulmonary fibrosis.

## Discussion

The evidence from recent years suggest that in addition to maintaining telomeres, non-telomeric roles for TERT may also be important in regulation of proliferation [14, 16], apoptosis [38], and mitochondrial function [17],. We previously demonstrated that telomerase and / or TERT are induced and required for BLM-induced pulmonary fibrosis, by comparing responses in TERT KO vs wild type mice [4, 25, 33]. However it is unclear if the deficient fibrotic response in TERT KO mice is solely due to ablation of TERT in the mesenchymal compartment, where TERT induction in the BLM model and in IPF lung fibroblasts has been observed. To elucidate this possibility, in this study, we established a floxed TERT mouse strain and further generated a Cre-inducible TERT CKO mouse line to conditionally knockout TERT gene specifically in type I collagen-expressing mesenchymal cells. Using this TERT CKO mouse line we established that TERT and its induction in mesenchymal/MLF was essential for pulmonary fibrosis since BLM-induced fibrosis was significantly attenuated in TERT CKO mice along with decreased telomerase activity in the lung tissue resulting from mesenchymal TERT deficiency. This was consistent with the previous finding that the induction of telomerase in lung fibroblasts is positively associated with IPF and other fibrotic interstitial lung diseases [33]. The involvement of telomerase induction in injury has also been reported in other studies. A rare but heterogeneous population of TERT-expressing cells, including cardiomyocytic, endothelial, fibroblastic and putative stem cells, is identified in mTERT promoter-drive GFP reporter mice. Cardiac injury results in 6.45-fold and 500-fold expansions of this mTERT-GFP cell population in the adult heart and within the “injury-zone”, respectively compared with sham-operated controls at 14 days following cryoinjury. Moreover, the identification of the TERT-expressing putative cardiac stem cells suggests that they represent a possible target for cardiac regeneration potential [40]. It is noteworthy that TERT deficiency in the current study was associated with reduced proliferation and increased differentiation, which would be consistent with the observation of TERT expression in less differentiated stem cell populations and their expansion in the cardiac injury study. A pivotal role of telomerase/TERT is also implicated in the allergic reactions in mice sensitized with IgE-specific TNP antibody followed by administration of TNP-OVA. IgE-mediated anaphylactic responses are largely attenuated in TERT KO mice with decreased number of mast cells in vivo [41]. However the demonstration of selective mesenchymal cell TERT deficiency is unique to the current study and revealed for the first time the importance of TERT induction in this select cell population in pulmonary fibrosis. Of note, TERT ablation in MLF was not complete after tamoxifen-induced Cre activation both in vitro and in vivo. There was still ~30% of residual TERT gene expression remaining in TERT CKO MLF, and about a 50% reduction in telomerase activity. These may be partly due to insufficient Cre-activation by tamoxifen and level of type I collagen expression present in the crossed mice TERT fl/fl,Cre+/-. The lesser inhibition of telomerase activity was likely due to the relative stability of the TERT protein vs. mRNA, thus even after the gene is disrupted persistence of the protein would still enable activity to be detected. Nevertheless, this degree of TERT/telomerase deficiency was sufficient to cause significant impairment of pulmonary fibrosis, indicative of the essential role of induced TERT in mesenchymal cells for fibrosis.

The BLM-induced rodent lung fibrosis model is the most frequently used animal model to investigate mechanisms and potential novel antifibrotic therapies for human IPF despite well-recognized shortcomings. However recent studies using genomics and gene set enrichment analysis suggest that there are commonalities between the BLM model and IPF with respect to disease relevant molecular alterations and translational gene markers. These findings suggest that the BLM model recapitulates many of the complex profibrotic responses in human IPF, and could be used to predict the pharmacological impact of treatment [42, 43]. Nevertheless the limitations of animal models need to be considered. While telomerase is significantly induced in lung fibroblasts from rodents with pulmonary fibrosis as well as a majority of patients with fibrotic interstitial lung diseases, including IPF [33], shortened telomeres in peripheral blood leukocytes have been reported in familial IPF and a minority of patients with sporadic IPF [12, 25, 27, 28, 33]. Moreover lung fibroblasts from animal models and most patients with IPF do not exhibit shortened telomeres, while shortened telomeres in late generation TERC deficient mice are not associated with increased susceptibility to BLM-induced pulmonary fibrosis [33]. The basis of this apparent discrepancy between shortened telomeres in peripheral blood leukocytes vs. lung fibroblasts and their significance for fibrosis is unclear, but may be related to the different functional significance of telomerase and/or telomere length in different cell types. Exploration of the significance of telomerase in the context of cell type is important for the elucidation of the differential pathophysiological role of telomerase in different cell types, for example, in epithelial versus mesenchymal cells during lung fibrosis. Accumulating evidence supports the emergence of an “apoptosis paradox” paradigm, suggesting two seemingly contrasting tendencies exist in IPF/UIP: increased apoptosis in epithelial cells, and decreased apoptosis in fibroblasts [44]. While induced telomerase is beneficial for epithelial cell repair attributed to improved survival and proliferation/regeneration, such induction in mesenchymal/MLF may be harmful to the tissue repair due to enhanced proliferation and persistence of fibroblasts resulting in an exuberant and pathological chronic fibrotic response. The findings in the current study revealed that it is the induced TERT/telomerase in mesenchymal cells that is important for BLM-induced pulmonary fibrosis. The role or importance of TERT in other cell types can be evaluated in future studies using these floxed TERT mice crossed to cell specific marker gene promoter driven Cre. The generation of this floxed TERT mouse makes it possible to study its cell context specific role in the particular cell type of interest in diverse animal models.

The increased number of fibroblasts and persistence of differentiated myofibroblasts are the key features during pulmonary fibrosis [45]. The impact of TERT deficiency in decreasing cell proliferation but increasing apoptosis rate in the collagen I-expressing MLF provides possible mechanisms for the impaired pulmonary fibrosis in TERT CKO animals. The results here suggested that TERT deficient MLF showed much lower proliferation rate and failed to respond to growth factor stimulation. This decreased proliferation ability was also seen in the MLF isolated from systemic TERT KO lungs after BLM treatment [25]. This role of TERT in cell proliferation has been reported also in other cells and tissues. BM cells from TERT KO mice show a limited cell expansion capacity and enlarged senescent morphology both in vitro and in vivo [41]. The epidermis of the transgenic mice with TERT overexpression in basal keratinocytes is highly sensitive to the mitogenic effects of phorbol esters with increased proliferation of basal keratinocytes [10]. The exogenously TERT transduced Dyskeratosis Congenital fibroblasts have a significantly extended lifespan, which is greater than three times that of controls in long-term cultures. These TERT-expressing fibroblasts display similar morphology to early passage normal fibroblasts by visual examination, however the extended fibroblast lifespan by transduction of TERT is not accompanied by telomere elongation [46, 47]. These findings suggest the promotion of proliferative potential and resistance to apoptosis by TERT may be responsible for the mechanism of increased number of mesenchymal cells or fibroblasts in pulmonary fibrosis.

Our data revealed that TERT deficient MLF had a higher apoptosis rate in comparison to control MLF, and these cells exhibited more susceptible to apoptosis induction, suggesting that TERT may play a key anti-apoptotic role. Telomerase is a known apoptosis alleviating factor. The knockdown of hTERT in scar fibroblasts by liposome-adenoviral transduction caused significantly increased apoptosis rate along with reduced telomerase activity and shortened telomere length [48]. In BLM-treated AEC II, the initially increased level of telomerase delays AEC II apoptosis, but the reduced telomerase at a later stage of treatment is associated with a significant increase in apoptosis rate [4, 49]. There may also be a time or stage dependent interaction between TERT induction and myofibroblast differentiation. Notably, the basal level of α-SMA in TERT deficient MLF was higher than that in control cells, even though the BLM caused induction of α-SMA was significantly reduced[39]. The induced TERT in fibrosis may be responsible for the cell proliferation, and lead to the increase in number of collagen-expressing fibroblasts, which are the precursors for the myofibroblasts. Thus the lack of TERT would result in a reduction in the precursor fibroblasts, eventually resulting in reduced myofibroblasts as reflected in lower α-SMA expression. This might be a potential mechanism by which myofibroblast differentiation was suppressed in BLM injury in TERT CKO mice. The evidence from previous and current studies shows TERT is transiently induced in this lung fibrosis model during day 7–14 after BLM treatment [4, 49], which precedes the peak of α-SMA expression or myofibroblast differentiation that is typically seen on 21 days post BLM treatment [4, 50].Thus the inhibitory effect of TERT deficiency in mesenchymal cell proliferation, survival and persistence may lead to less severe fibrosis in TERT CKO mice.
